# TaqMan® and HRM approaches for SNP genotyping in genetic traceability of musts and wines

**DOI:** 10.1016/j.crfs.2024.100707

**Published:** 2024-02-22

**Authors:** Amedeo Moine, Paolo Boccacci, Camilla De Paolis, Luca Rolle, Giorgio Gambino

**Affiliations:** a Institute for Sustainable Plant Protection, National Research Council (IPSP-CNR), Strada Delle Cacce 73, 10135, Torino, Italy; b Department of Agricultural, Forest and Food Sciences, University of Turin, Largo Braccini 2, 10095, Grugliasco, TO, Italy; c Interdepartmental Centre for Grapevines and Wine Sciences, University of Turin, Corso Enotria 2/C, 12051, Alba, CN, Italy

**Keywords:** Genetic traceability, Grapevine, Musts, Wines, SNPs

## Abstract

The fight against fraud in the wine sector requires continuous improvements and validations of new technologies applicable to musts and wines. Starting from published data from the Vitis18kSNP array, a series of new specific single nucleotide polymorphism (SNP) markers have been identified for some important north-western Italian cultivars, such as Barbera, Dolcetto and Arneis (*Vitis vinifera* L.), used in the production of high-quality wines under Protected Denomination of Origin. A pair of new SNP markers for each grape variety were selected and validated using two real-time PCR techniques: TaqMan® genotyping assays and high-resolution melting analysis (HRM). The TaqMan® assay has proven to be more reliable and repeatable than HRM analysis because despite being an economical and versatile technique for the detection of different types of genomic mutations (SNPs, insertions or deletions), HRM has shown limitations in the presence of poor-quality DNA extracted from musts and wines. TaqMan® assays have successfully identified Barbera, Dolcetto and Arneis in their respective musts and experimental wines, and with good efficiency in commercial wines. Marked differences between genotypes were observed, varietal identification in Dolcetto-based musts/wines was more efficient than that in Arneis-based wines. Therefore, the TaqMan® assay has considerable potential for varietal identification in wines and the procedure described in the present work can be easily adapted to all wines with adequate setup of DNA extraction methods that should be adapted to different wines.

## Highlights

• Single nucleotide polymorphism (SNP) markers specific for different grape varieties.
• SNP markers detected by TaqMan® genotyping assays and HRM analysis.
• TaqMan® assays have successfully identified experimental and commercial wines.
• SNP genotyping are promising method for varietal identification in wine.

## Graphical abstract

**Figure:**
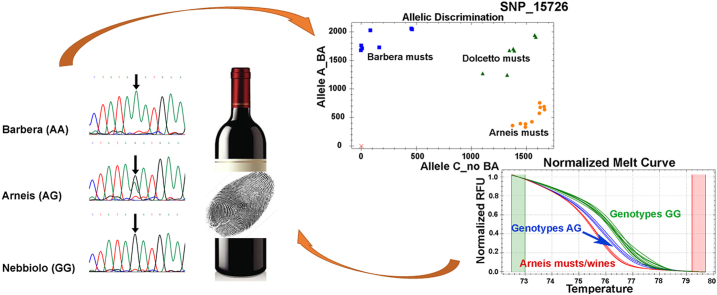

## 1 Introduction

Authenticity, safety, and traceability of high commercial value wines produced under Protected Denomination of Origin (PDO) are major concerns for markets and consumers since wine is susceptible to fraud, adulteration and mislabelling. The addition of water, glycerol, alcohol, dyes, sweeteners, flavourings, unapproved sugar additions, and acidity modifications (Schlesier et al., 2009), as well as the use of wines from grape varieties different from those allowed, are examples of adulteration that can change the value of a high-quality wine. In this scenario, the European Union has created specific rules for wine traceability to protect both consumers and winemakers (Regulation EC No. 1151/2012 and subsequent amendments).

A wide range of analytical approaches for wine traceability and authentication, such as chemical analysis of proteins, volatile compounds, amino acids, polyphenols, anthocyanins, and minerals have been developed and evaluated (Moreno Arribas et al., 1999; Versari et al., 2014). However, because chemical compositions can be modified by winemaking procedures, farming techniques and environmental factors, these approaches are not always accurate (Villano et al., 2017). Since DNA is a stable molecule, DNA-based techniques are considered more reliable for food authentication (Martins-Lopes et al., 2013; Scarano and Rao, 2014; Catalano et al., 2016). Single sequence repeats (SSRs) are considered the most common markers for grapevine fingerprinting and are characterized by locus-specific polymorphism. As a result, many studies have used SSRs for grape identification in mono- and multi-varietal musts and wines (Faria et al., 2000; Siret et al., 2002; Boccacci et al., 2012; Pereira et al., 2012; Recupero et al., 2013; Catalano et al., 2016; Zambianchi et al., 2021, 2022). However, when considering SSR genotyping in wine, polymerase chain reaction (PCR) amplification results are often inaccurate due to DNA degradation during alcoholic fermentation (low quality and quantity) and for the presence of PCR inhibitors such as polyphenols, proteins and polysaccharides (Vignani et al., 2019).

Single nucleotide polymorphisms (SNPs) have been proposed as new molecular markers for grapevine fingerprinting and have become a valid alternative to SSR genotyping in musts and wines (Cabezas et al., 2011). SNPs are the most abundant markers in the genome, purely biallelic with a low mutation rate, and can be detected and amplified by PCR in low-quality fragmented DNA (Cabezas et al., 2011; Catalano et al., 2016; Fanelli et al., 2021). Unlike SSR markers, many SNPs are needed for varietal identification; however, the progressive reduction in sequencing and data analysis over the years has allowed the identification of several mutations and polymorphisms between different grapevine cultivars (Emanuelli et al., 2013; Mercati et al., 2016; Gambino et al., 2017; De Lorenzis et al., 2019). Recent reports have explored the potential of SNP genotyping for must and wine traceability using qPCR coupled with a specific TaqMan® assay protocol (Catalano et al., 2016; Boccacci et al., 2020; Gambino et al., 2022; Song et al., 2024) or high-resolution melting (HRM) technology (Pereira and Martins-Lopes, 2015; Pereira et al., 2017,^2018^). The SNP TaqMan® genotyping assay provides significant technological advantages, including a single enzymatic step, flexible primer location in the region surrounding the SNP site, high sensitivity and specificity in DNA detection using labelled probes, and reduced analysis time (Boccacci et al., 2020). HRM is a versatile post-PCR method that can be used for the identification of SNPs, SSRs and other mutations, such as insertions and deletions (INDELs), and it can be used in the field of food traceability (Mackay et al., 2008; Xanthopoulou et al., 2014; Merkouropoulos et al., 2016; Villano et al., 2017; Pereira et al., 2017; Barrias et al., 2023). HRM technology entails qPCR amplification in the presence of a saturation dye and subsequent melting of the amplicons by progressively increasing the temperature. The unique shape of the melting curve depends on the length, sequence, GC content and melting temperature of the amplicon (Simko, 2016). Primer design, PCR reagents, DNA (quality and quantity), amplicon length, multiple SNPs in the same DNA region and dye selection are all important factors that influence the success of HRM genotyping assays (Pereira et al., 2017). In wine, HRM has been performed on amplicons containing at least four SNPs and one INDEL in the same DNA region (Pereira et al., 2017). Nevertheless, there are no studies in the literature wherein HRM has been applied to a single SNP for varietal identification in wines.

Starting from previous experience in our laboratory in which an efficient traceability method in Nebbiolo (*Vitis vinifera* L.) wines based on SNPs and TaqMan® probes was developed (Boccacci et al., 2020; Gambino et al., 2022; Song et al., 2024), we extended and implemented this procedure to other important grapevine genotypes used to produce high-quality wines. Barbera and Dolcetto (*V. vinifera*) are two of the most important traditionally cultivated varieties in Piedmont (northwest Italy) and are the major and third regional black grapes, respectively, that contribute to fine PDO wines. Arneis (*V. vinifera*), an Italian autochthonous white grape cultivar grown mainly in Piedmont, is used to produce homonymous white wines. The wines derived from these genotypes are an excellent starting point for the development of a genetic traceability system that can be extended to different wines.

The aim of this study was to develop and validate effective assays for the genetic traceability of musts and wines typical of north-western Italy produced from Barbera, Dolcetto and Arneis grapes. We identified cultivar-specific SNPs starting from available databases produced using the Vitis18kSNP array (https://urgi.versailles.inra.fr/Species/Vitis/GrapeReSeq_Illumina_20K). This array, containing more than 18,000 SNPs, was produced using sequencing data from different genotypes of *V. vinifera* and other *Vitis* species and it has already been used to study the genetic relationships between cultivated and wild grapevine germplasm (Laucou et al., 2018; De Lorenzis et al., 2019). In addition, we implemented rapid SNP assays for varietal authentication in wine by comparing TaqMan® and HRM approaches to establish the best SNP-based traceability procedure. The proposed and validated protocol can be easily adapted and used for varietal identification in any wine.

## 2 Methods

### 2.1 Plant material and SNP identification

DNA from Barbera, Dolcetto and Arneis was extracted from young leaves using a NucleoSpin® Plant Kit (Macherey-Nagel, Düren, Germany) following the manufacturer’s instructions. In addition to ampelographic observations, the plants were genotyped using six SSR markers (This et al., 2004; Ruffa et al., 2016) to confirm the identity of the three cultivars.

We collected and processed all SNP data available in the literature by analysing several grapevine genotypes using the Vitis18kSNP array (Illumina, Inc., San Diego, CA, USA). In addition to SNP data obtained in cultivars typical of northwestern Italy (Boccacci et al., 2020; Raimondi et al., 2020), we processed the data from six available SNP databases (Laucou et al., 2018; De Lorenzis et al., 2019; D’Onofrio et al., 2021; D’Onofrio, 2020; Crespan et al., 2020; Crespan et al., 2021) for a total number of 1857 genotypes (Table S1). The SNP data were subjected to several filtering steps following the previous indications (Boccacci et al., 2020): i) development of an overall database without redundant genotypes; ii) removal of SNPs with missing data even in a single genotype; iii) removal of SNPs with heterozygous allelic profile and selection of SNPs with a homozygous allelic profile in the cultivar of interest; iv) selection of SNPs with a “homozygous alternative” allelic profile to the cultivar of interest in other cultivars, in order to facilitate varietal identification in unknown wine samples. The two best SNPs respecting these parameters specific for Barbera, Dolcetto and Arneis were validated by PCR amplification and Sanger sequencing, as reported by Boccacci et al. (2020). The primers used are reported in Table S2.

### 2.2 Experimental vinification and commercial wines

A total of 100 kg of grapes for each true-to-type Arneis, Barbera and Dolcetto cultivars were used to produce the experimental wines.

Arneis grapes, after harvesting, were placed in a thermo-controlled room at 10 °C overnight to use refrigerated grapes. After this cool time these were destemmed and crushed in a TEMA de-stemmer–crusher (Enoveneta, Piazzola Sul Brenta, Italy). Then the crushed grapes were pressed using a PMA 4 pneumatic press (Velo SpA, Altivole, Italy), reaching the maximum pressure of 1.0 bar. After mashing, a 24-h cold static clarification was carried out on grape juice, using 2 g/hL of pectolytic enzyme (Lallzyme cuvée blanc, Lallemand Inc., Montreal, Canada). Successively must was racked and finally inoculated for the alcoholic fermentation, with 20 g/hL of *Saccharomyces cerevisiae* active dry yeast (Fermol Chardonnay, AEB Group, San Polo, Italy). During the alcoholic fermentation, the temperature (18/±1 °C) and the sugar decrease were daily controlled and two additions (one at the beginning and the second at 1/3 of fermentation process) of 20 g/hL of nutrients (Fermaid E, Lallemand Inc.) corresponding to a total increase of 56 mg/L yeast assimilable nitrogen (YAN) were done. At the end of alcoholic fermentation, the wine was racked and 50 mg/L of SO_2_ added.

Barbera and Dolcetto grapes were destemmed and crushed (Enoveneta, Piazzola Sul Brenta, Italy). The mash was placed into a CO_2_ saturated inox tank and inoculated for the alcoholic fermentation with 20 g/hL of *Saccharomyces cerevisiae* active dry yeast (Lalvin BRL 97, Lallemand Inc.). During the alcoholic fermentation, the temperature (26/±1 °C) and the sugar decrease were daily controlled. As in the white vinification two addition of nutrients (Fermaid E, Lallemand Inc.) have been done. Moreover, two punch-down per day were carried out in the first three days and subsequently two pumping-over were done until the end of maceration, which lasted 8 days. Later, the pomace cap was pressed (Velo SpA, Altivole, Italy) and the pressed wine was added to the free-run part. Then 1 g/hL of lactic bacteria *Oenococcus oeni* VP41 MBR ML (Lallemand Inc.) was added to inoculate the malolactic fermentation. At the end, the wines were racked and 50 mg/L of SO_2_ added. The wines, both red and white, were stored at 0 °C for two weeks for cold stabilization and finally filtered and bottled.

The red wine samples (500 mL) were collected in five different steps: after crushing (M1), at the end of maceration (M2), after alcoholic fermentation (M3), after the malolactic fermentation (M4), and once bottled (W). Instead, Arneis samples were only collected in three steps: on the juice after mashing (M1), at the end of alcoholic fermentation (M3) and after bottling (W). This because in the white winemaking usually there is no maceration, and the malolactic fermentation is not performed. For all samples, 50 mL aliquots were stored at −20 °C until DNA extraction.

Commercial wines from Barbera (Barbera d' Alba, 2020, 2021 and 2022), Dolcetto (Dolcetto d' Alba, 2021 and 2022) and Arneis (Roero Arneis 2022) grapes were provided by Enocontrol Scarl (Alba, Italy). Before DNA isolation, each wine was homogenised by inverting the bottle, and 50 mL aliquots were stored at −20 °C until DNA extraction.

### 2.3 DNA extraction and quantification

Total DNA extraction from each must and wine was performed following the Cetyltrimethylammonium Bromide (CTAB)-based protocol described by Gambino et al. (2022). Briefly, 50 mL of musts and wines frozen at −20 °C for at least 15 days was centrifuged (4000 *g*, 1 h, 4 °C). About 200–300 mg of each must pellet and the wine pellet collected from 50 mL were dissolved in 5 mL of buffer containing 20 mM EDTA (pH 8.0), 1.4 M NaCl, 1 M Tris–HCl (pH 8.0), 3% CTAB, 1% β-mercaptoethanol and incubated for 1 h at 65 °C. A volume of 5 mL of chloroform:isoamyl alcohol (C:I 24:1) was added and centrifuged at 8000 *g* for 10 min at 4 °C. The supernatant with 0.1 volume of prewarmed (65 °C) 10% CTAB was purified with 1 volume of C:I. Two volumes of cold ethanol were added to the supernatant and stored overnight at −25 °C. The precipitated DNA (10,000 *g*, 30 min, 4 °C) was suspended in Tris-EDTA (TE) buffer (250 μL) and incubated with the addition of proteinase K (20 μL, 20 mg/mL) for 30 min at 48 °C. The sample was purified with 1 volume of phenol:chloroform:isoamyl alcohol (25:24:1) and centrifuged (11,000 *g*, 15 min, 4 °C). Two volumes of cold ethanol and 2.5 mol/L of ammonium acetate were added to the supernatant and stored for 2 h at −25 °C. The pellet obtained after centrifugation (20,000 *g*, 30 min, 4 °C) was washed with 500 μL 70% ethanol and dissolved in 100 μL sterile water. Final purification was performed with the NucleoSpin® Plant Kit (Macherey-Nagel, Düren, Germany) according to the manufacturer’s instructions. The final elution was performed in 45 μL PE buffer.

Total DNA was preliminary evaluated using a NanoDrop 1000 spectrophotometer (Thermo Fisher Scientific, Waltham, MA, USA) by determining the spectrophotometric absorbance of the samples at 230, 260 and 280 nm. Grapevine DNA was measured by qPCR amplification of the 9-*cis*-epoxycarotenoid dioxygenase gene (*VvNCED2*) using the TaqMan® FAM-labelled probe and methods reported by Savazzini and Martinelli (2006). The qPCR reaction was performed with a reaction volume of 10 μL, containing 2.5 μL of extracted DNA, 5 μL of TaqMan® Environmental Master Mix 2.0 (Thermo Fisher Scientific), 0.3 μM of each primer and 0.2 μM of the FAM probe. Amplification, carried out using a CFX96 Detection System (Bio-Rad Laboratories, Inc., Hercules, CA, USA) was performed as follows: initial denaturation step at 95 °C for 10 min, 55 cycles of 95 °C for 15 s, and 60 °C for 1 min. The grapevine DNA concentration was determined by plotting the Ct values obtained from the DNA of the samples with the standard curve of the *VvNCED2* produced with serial dilutions of DNA extracted from leaves. All samples were analysed in triplicate.

### 2.4 SNP genotyping protocols and data analysis

We compared two genotyping protocols (TaqMan® probes and HRM analysis) to identify the cultivar-specific SNPs in musts and wine of Barbera, Dolcetto and Arneis. For each cultivar-specific SNP, primers and TaqMan® probes were designed using Primer Express version 3.0 (Thermo Fisher Scientific) (Table S3). The amplification reaction was performed in triplicate in a reaction volume of 10 μL, containing 2.5 μL of DNA, 5 μL TaqMan® Environmental Master Mix 2.0 (Thermo Fisher Scientific) and 0.25 μL of 40X TaqMan® SNP Genotyping Assay mix (containing pre-mixed forward and reverse primers, VIC probe and FAM probe). The amplification profile used was the same as reported for the *VvNCED2* probe. The threshold position was automatically calculated using Bio-Rad CFX Manager 3.1 software (Bio-Rad Laboratories, Inc.), and allelic discrimination plots were performed using the CFX96 Detection System.

In the HRM protocol, we used the same primers reported for TaqMan® assays (Table S3) and the amplification reaction was carried out in triplicate with a total volume of 10 μL, containing 2.5 μL of DNA, 5 μL MeltDoctor™ HRM Master Mix (Thermo Fisher Scientific) and 0.2 μM of each primer. Amplification, using a CFX96 Detection System, was performed as follow: initial denaturation step at 95 °C for 10 min, 55 cycles of 95 °C for 30 s, 60 °C (58 °C for SNP_87) for 30 s and 72 °C for 30 s. Melting curves were generated over a 60–95 °C range with an increment of 0.1 °C every 5 s. During the incremental melting step, fluorescence data were continuously acquired and analysed using High-Resolution Melt Software v3.0.1 (Bio-Rad Laboratories, Inc.).

PCR inhibitors in the extracted DNA were determined by adding TaqMan® Exogenous Internal Positive Control (EIPC) reagents (Thermo Fisher Scientific) to the qPCR mix. The amplification reaction in a final volume of 10 μL contained 2.5 μL genomic DNA, 5 μL TaqMan® Environmental Master Mix 2.0 (Thermo Fisher Scientific), 0.2 μL EIPC DNA and 1 μL EIPC mix (containing pre-mixed forward, reverse primers and VIC probe specific for EIPC). The amplification profile used was the same as reported above for TaqMan® assays. PCR inhibition was calculated from a calibration curve with serial dilution of EIPC, assuming 100% amplification efficiency of EIPC in samples containing DNA extracted from leaves.

Statistical analyses were carried out using a one-way analysis of variance (SPSS Version 22). The differences among the treatments were indicated with different letters using Tukey’s post-hoc test at p-value ≤0.05.

## 3 Results and discussion

### 3.1 Identification of cultivar-specific SNPs

In recent years, in addition to SSRs, which have historically been considered the markers of choice for cultivar identification in grapevine (This et al., 2004; http://www.eu-vitis.de/index.php; http://www.vivc.de), SNPs are becoming highly performing markers and are widely used in grapevine genotyping studies (Myles et al., 2010; Laucou et al., 2018; Raimondi et al., 2020; D'Onofrio et al., 2021). Due to their characteristics, SNPs have been very useful in varietal identification in musts and wine for genetic traceability studies (Catalano et al., 2016; Pereira et al., 2017; Boccacci et al., 2020; Carrara et al., 2023). However, for SNP detection in wine, it is not effective to use arrays, such as the Vitis18kSNP array (Illumina) or high throughput sequencing, due to the low quality of the extracted DNA, while high-performance qPCR-based detection systems, which work effectively even with traces of DNA, are recommended. Accordingly, the identification of a minimum number of specific and unique SNP markers (ideally 1 or 2) for each cultivar is a fundamental prerequisite for the practical application of SNPs in molecular traceability in the wine field.

Based on seven studies (Laucou et al., 2018; De Lorenzis et al., 2019; D’Onofrio et al., 2021; D’Onofrio, 2020; Crespan et al., 2020; Crespan et al., 2021; Raimondi et al., 2020) that published SNP profiles of many grapevine genotypes using the Vitis18kSNP array, we produced an overall database to identify cultivar-specific SNPs. A total of 1857 accessions analysed in these seven works corresponded to 1493 unique non-redundant genotypes. Among them, 4408 of the 18,071 SNP markers analysed with the Vitis18kSNP array failed or showed an unclear hybridisation signal and were thus discarded. The remaining 13,663 SNPs were further filtered for each cultivar. For example, we selected SNPs that were homozygous and without polymorphisms within the Arneis accession present in the database but none of these SNPs were specific to Arneis. Therefore, we selected SNPs showing a profile homozygous alternative to Arneis in the largest number of non-Arneis cultivars, because they are potentially more discriminating in SNP genotyping assays (Boccacci et al., 2020). Within this group of markers, two SNPs located on different chromosomes were therefore extrapolated (Table S1). The combination of which was unique in Arneis (SNP_16408, SNP_6647) and sufficient to uniquely identify Arneis among the 1493 genotypes present in the overall SNP database. Following the same procedure, we identified two SNP markers whose allelic combination was specific for Barbera (SNP_15726 and SNP_3356); the same was done for Dolcetto (SNP_1722 and SNP_87) (Table S1).

The six selected SNPs were validated by Sanger sequencing confirming the hybridisation results (Fig. S1). In addition, for each cultivar, clone selections officially registered in the Italian National Register of Grape Varieties (http://catalogoviti.politicheagricole.it/catalogo.php) were collected from nurseries, and we confirmed that all accessions had the same allelic profiles reported in the SNP database, suggesting that these SNPs were very robust markers specific for Arneis, Barbera or Dolcetto. This cultivar-specific SNP selection methodology, first used in Nebbiolo (Boccacci et al., 2020), has now been successfully confirmed for three other varieties, and can be extended very easily to any genotype analysed with the Vitis18kSNP array. This approach should be more effective for identifying 1 or 2 cultivar-specific SNPs that can be analysed in wine using qPCR techniques, compared, for example, to identifying mutations in genes such as UDP-Glucose:Flavonoid 3-O-Glucosyltransferase (Pereira and Martins-Lopes, 2015) or flavanone 3-hydroxylase and the leucoanthocyanidin dioxygenase gene (Gomes et al., 2018), which would require a large unavailable database and would be complex to produce considering the high number of grapevine genotypes. The specificity of the SNPs identified in the output of the Vitis18kSNP array was validated by the large number of unique genotypes analysed thus far in seven previously published studies (1493), which included all major wine varieties that could potentially be used for fraudulent blends. However, if this method of identifying varietal SNPs is useful for targeted analyses of wines, it is not particularly suitable for traditional grapevine fingerprinting, as it is more appropriate to use arrays (Myles et al., 2010; Laucou et al., 2018) or a selection of more polymorphic SNPs (Cabezas et al., 2011).

### 3.2 Genotyping assays using HRM and TaqMan® approaches

High-efficiency analysis of SNPs can occur using two qPCR approaches: HRM and TaqMan® probes. HRM represents a simple and low-cost technique for the identification of genomic mutations (SNPs, INDELs), and it has been successfully applied for the authenticity of olive oil and wine (Pereira et al., 2017,^2018^). Compared to the TaqMan® technique, HRM is more versatile for detecting different mutations (including INDELs) and is more suitable for difficult genomic regions, such as repeat sequences in nucleotides surrounding SNPs, where it is difficult to design TaqMan® probes. However, in previous studies (Pereira et al., 2017, 2018), HRM was applied to amplicons containing several SNPs and INDELS in each fragment, facilitating the genotype identification using the difference in melting curves. In our experiment, with only one SNP available in each amplicon, the efficiency of the HRM technique should be evaluated and compared with the TaqMan® approach which proved to be very efficient for the detection of a single SNP in must and wine (Catalano et al., 2016; Boccacci et al., 2020; Gambino et al., 2022; Song et al., 2024).

**Fig. 1:**
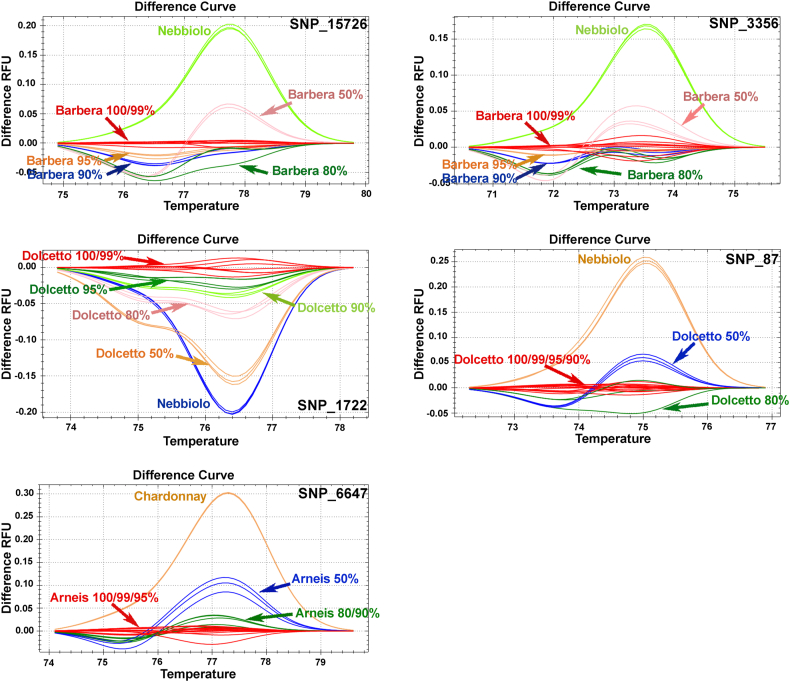
Detection limit of HRM genotyping assays in mixtures of DNA extracted from leaves. Samples were grouped based on the melting curve shape shown in the difference plots. SNP_3356, SNP_15726 (Barbera-specific), SNP_6647 (Arneis-specific), SNP_1722 and SNP_87 (Dolcetto-specific). Increasing levels of non-target DNA (1–50%) were mixed with the target DNA. For SNP_3356 and SNP_15726, Nebbiolo was used as the non-target and Barbera as the target genotype; for SNP_1722 and SNP_87, Nebbiolo was used as the non-target and Dolcetto as the target genotype; for SNP_6647, Chardonnay was used as the non-target and Arneis as the target genotype. The detection limit was determined using triplicates of each sample. Genotypes were assigned using a cut-off confidence value of 95%.

The six SNP loci for Arneis, Barbera and Dolcetto were analysed by HRM using specific primers (Table S3) on DNA extracted from leaves of true-to-type plants. The results confirmed the efficiency of HRM in SNP genotyping and cultivar specificity, with only five out of six selected SNPs (Fig. S2). SNP_16408 (specific for Arneis) showed some problems in the melting difference plot with incorrect distinction between homozygous and heterozygous genotypes (Fig. S2). It is likely that this genomic region is not optimal for accurate melting curve analysis, as it presents some problems (Fig. S3), and the marker is not efficient for genotyping with HRM. In addition, to assess the limits of the technique for detecting blends, we produced an artificial mix of genomic DNA, increasing the levels of non-target DNA in the target DNA (Fig. 1). Non-Barbera DNA was mixed with Barbera DNA (from 0.1% to 50% v/v of contamination), and SNP_15726 and SNP_3356 were analysed using HRM. The same procedure was carried out for SNPs specific to Arneis (excluding SNP_16408) and Dolcetto. Data obtained from HRM showed that the detection limit of non-specific cultivars in the DNA mixture was 20% for SNP_87, 10% for SNP_6647, and 5% for SNP_1722, SNP_15726 and SNP_3356 (Fig. 1).

**Fig. 2:**
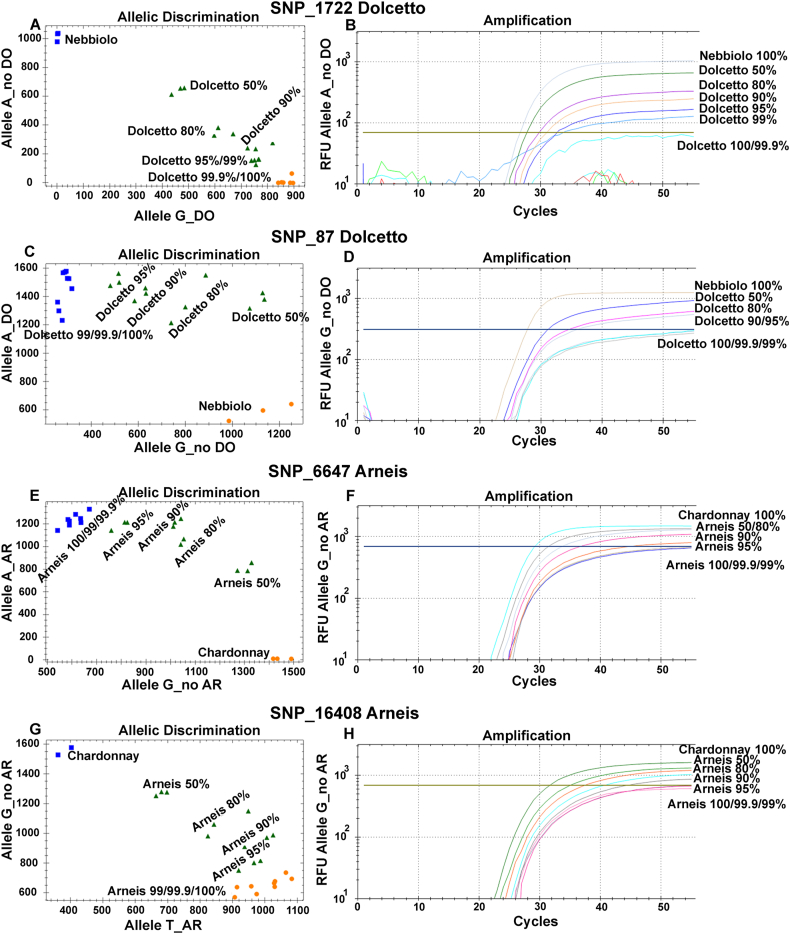
Detection limit of TaqMan® SNP_1722, SNP_87, SNP_6647 and SNP_16408 genotyping assays in mixtures of DNA extracted from leaves. Allelic discrimination plots (A, C, E, G) and relative fluorescence unit (RFU) of TaqMan® probes for non-specific alleles (B, D, F, H). Nebbiolo and Chardonnay DNA were used as non-specific genotypes in the SNP assays for Dolcetto (A–D) and Arneis (E–H), respectively. Increasing levels of Nebbiolo DNA (from 0.1 to 50%) were mixed with Dolcetto DNA (A–D). The yellow line (B) and blue line in the amplification plot (D) indicate the RFU level of 100% Dolcetto, above which it was possible to detect contamination of non-Dolcetto DNA. Increasing levels of Chardonnay DNA (from 0.1 to 50%) were mixed with Arneis DNA (E–H). The blue line (F) and the yellow line in the amplification plot (H) indicates the RFU level of 100% Arneis, above which it was possible to detect contamination of non-Arneis DNA. For each SNP assay, the detection limits of non-specific DNA mixed in Dolcetto (A–D) or Arneis (E–H) DNA were determined using three replicates of each sample. (For interpretation of the references to colour in this figure legend, the reader is referred to the Web version of this article.)

The same primers used for detection in HRM of the six selected SNPs were applied in the TaqMan® approach in addition to specific probes labelled with FAM or VIC fluorescent dyes (Table S3). The TaqMan® discrimination plots confirmed the efficiency of the technique for SNP detection (Fig. S4) for all six SNP loci, including SNP_16408, showing a problem in HRM. The detection limit of non-specific cultivars in the artificial DNA mixture was 1% for SNP_1722 and 5% for the other five SNP genotyping assays (Fig. 2 and Fig. S5). Our data support the potentiality and sensitivity of SNP genotyping using TaqMan® probes, confirming a detection limit of 1% in the discrimination of DNA in extract mixtures for SNP_1722, as previously reported for SNPs specific to Nebbiolo (Boccacci et al., 2020), which is the lowest level described in the literature to date (Catalano et al., 2016; Siret et al., 2002). For the other five SNPs, the detection limits stand at 5%, but at levels higher than those referable, for example, to SSR markers. These data also highlight that the efficiency of the technique is closely linked to the genomic region in which the SNP marker is located, the surrounding sequences and the possibility of designing excellent quality primers and probes influence the performance of the TaqMan® approach and consequently not all SNPs may be suitable for varietal identification in complex matrices such as musts and wines. In addition, HRM analyses appear less performing compared to TaqMan® probes in the same SNP loci and using the same primers, with a clear reduction in the limit of detection for non-specific DNA in a mixture (in 4 out of 6 SNPs) and with incorrect distinction between homozygous and heterozygous genotypes of SNP_16408.

### 3.3 SNP genotyping in experimental musts and wines

Experimental musts/wines of Arneis, Barbera and Dolcetto obtained from true-to-type grapes were collected from five time points for red varieties (mashing_M1, end of maceration_M2, end of AF_M3, end of MLF_M4 and wine_W), considered the most impacting time points for DNA extraction during the winemaking process, as previously observed in Nebbiolo (Boccacci et al., 2020), and from three time points for Arneis (mashing_M1, end of AF_M3, wine_W). DNA from all samples was extracted using a CTAB-based method that has proven to be very effective in Nebbiolo wines (Gambino et al., 2022), and DNA was obtained from all time points with decreasing quantity and quality from musts to finished wine, as expected (Table 1). Considering the well-known problems in the spectrophotometry quantification of DNA extracted from musts/wines (Savazzini and Martinelli, 2006; Vignani et al., 2019; Boccacci et al., 2020; Gambino et al., 2022), due to the presence of yeast contamination, DNA degradation and the interference of other compounds, such as phenol used in DNA purification, the *VvNCED2* TaqMan® probe (Fig. S6) was used for a more specific quantification of grapevine DNA (Savazzini and Martinelli, 2006; Boccacci et al., 2020; Gambino et al., 2022). In musts/wines of Dolcetto and Barbera, the grapevine DNA varies from 3978 pg/μL in M1 to 0.28 pg/μL in W with comparable values between the two varieties (Table 1). The recovery of grapevine DNA after malolactic fermentation (M4) was significantly reduced, confirming the data reported in Nebbiolo (Boccacci et al., 2020). In musts/wines of Arneis, the M3 values were much lower than in Dolcetto and Barbera at the same collection time point, by a factor of 100, as well as in experimental wine by about 10 times (Table 1), but at this collection time point, the data were less significant, given that we are very close to the detection limit of the *VvNCED2*-based technique for wines (Boccacci et al., 2020). This difference in DNA extractability from red wines, such as Barbera and Dolcetto, to white wine such as Arneis, may be linked to the different mashing and clarification operation to which the different wines are subjected.

**Table 1:** DNA quantity and quality extracted from Barbera (_B), Dolcetto (_D) and Arneis (_A) musts (M) and wines (W) collected during five and three (for Arneis) experimental winemaking steps, and from commercial wines. Purity and yield measured by NanoDrop; yield evaluated by a standard curve with FAM-labelled endogenous gene *VvNCED2*. Data are means ± SDs of three replicates and are expressed as ng/μl of DNA eluted from the NucleoSpin® Plant Kit.

Must/Wine	Description	Spectrophotometric quantification			*VvNCED2* quantification DNA yield (ng/μl)
		DNA yield (ng/μl)	A_260_:A_280_	A_260_:A_230_	
**M1_B**	mashing	322.65 ± 44.49	2.08 ± 0.02	1.91 ± 0.11	3978.20 ± 678.45
**M2_B**	end maceration	391.15 ± 99.20	2.16 ± 0.01	2.39 ± 0.05	519.02 ± 5.04
**M3_B**	after AF*	9.38 ± 4.69	1.83 ± 0.14	0.91 ± 0.23	15.50 ± 6.05
**M4_B**	after MLF**	5.55 ± 0.49	1.90 ± 0.21	0.60 ± 0.05	0.28 ± 0.12
**W_B**	wine	4.40 ± 0.48	1.83 ± 0.18	0.67 ± 0.02	0.39 ± 0.55

**M1_D**	mashing	264.30 ± 55.14	2.07 ± 0.04	1.78 ± 0.24	3649.80 ± 451.2
**M2_D**	end maceration	130.90 ± 65.93	2.08 ± 0.03	1.74 ± 0.06	1134.61 ± 359.77
**M3_D**	after AF*	4.20 ± 0.14	1.64 ± 0.06	0.72 ± 0.04	5.88 ± 2.86
**M4_D**	after MLF**	7.30 ± 0.42	1.68 ± 0.09	0.89 ± 0.08	1.87 ± 1.15
**W_D**	wine	3.80 ± 0.28	1.54 ± 0.07	0.72 ± 0.02	0.34 ± 0.14

**M1_A**	mashing	377.40 ± 17.96	2.10 ± 0.01	2.12 ± 0.02	6829.81 ± 2683.5
**M3_A**	after AF*	6.25 ± 0.78	1.77 ± 0.10	0.61 ± 0.08	0.18 ± 0.25
**W_A**	wine	4.15 ± 0.21	1.70 ± 0.27	0.60 ± 0.01	0.04 ± 0.01

**Commercial wines**	Barbera 2020	9.10 ± 3.82	1.47 ± 0.04	0.46 ± 0.42	0.06 ± 0.11
	Barbera 2021	5.05 ± 0.49	1.60 ± 0.08	0.45 ± 0.23	–
	Barbera 2022_a	3.92 ± 0.37	1.88 ± 0.00	0.46 ± 0.03	1.06 ± 0.36
	Barbera 2022_b	3.15 ± 0.11	1.90 ± 0.22	0.63 ± 0.07	1.56 ± 0.43
	Dolcetto 2021_a	4.75 ± 0.07	1.50 ± 0.04	0.66 ± 0.00	0.69 ± 0.25
	Dolcetto 2021_b	6.20 ± 0.52	1.60 ± 0.17	0.63 ± 0.20	0.14 ± 0.20
	Dolcetto 2022_a	42.20 ± 0.42	1.93 ± 0.03	1.92 ± 0.06	7.64 ± 2.45
	Dolcetto 2022_b	9.17 ± 0.16	1.96 ± 0.00	1.10 ± 0.01	–
	Dolcetto 2022_c	32.03 ± 6.80	1.94 ± 0.02	0.79 ± 0.15	–
	Arneis 2022_a	5.80 ± 0.95	1.69 ± 0.03	0.76 ± 0.02	–
	Arneis 2022_b	5.40 ± 0.28	1.52 ± 0.13	0.72 ± 0.05	–
	Arneis 2022_c	504.28 ± 125.40	2.17 ± 0.02	2.34 ± 0.02	0.14 ± 0.11

*AF= alcoholic fermentation.

**MLF=malolactic fermentation.

**Fig. 3:**
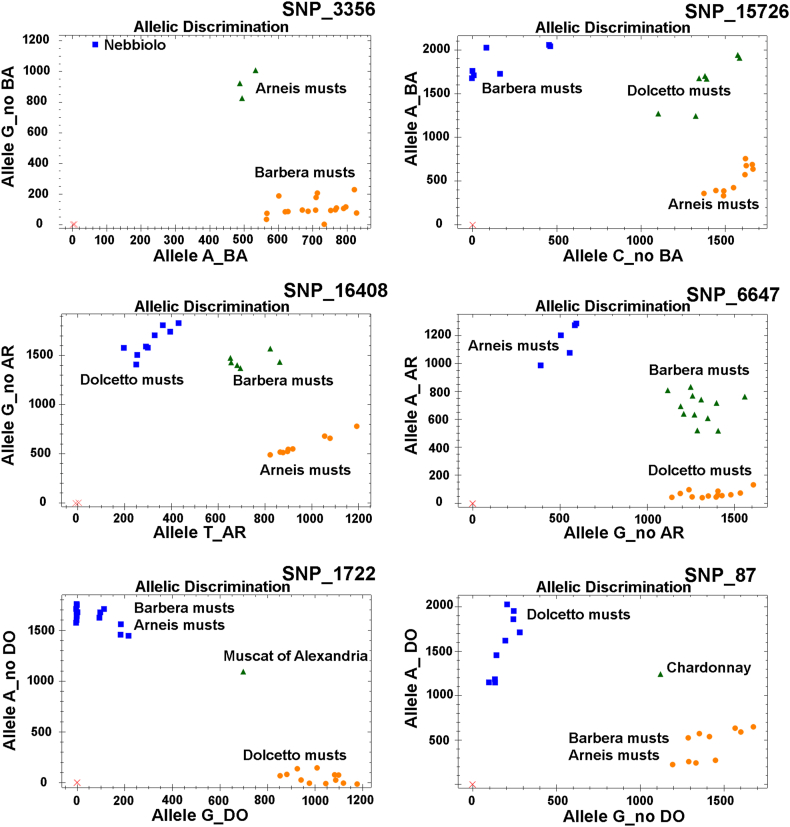
Output of TaqMan® SNP_3356, SNP_15726 (Barbera-specific), SNP_16408, SNP_6647 (Arneis-specific), SNP_1722 and SNP_87 (Dolcetto-specific) genotyping assays. Blue squares and orange points correspond to homozygous genotypes; green triangles are controls for heterozygous genotypes. DNA was extracted from experimental musts and wines of Barbera, Dolcetto and Arneis (details in Table 2). (For interpretation of the references to colour in this figure legend, the reader is referred to the Web version of this article.)

The extracted DNA was then analysed with the six selected SNPs using HRM and TaqMan® approaches. Each SNP was analysed in all types of wines and musts; for SNP_3356 and SNP_15726 (Barbera-specific), the Barbera musts/wines represented the target controls and the Dolcetto and Arneis musts/wines represented the non-target controls. For SNP_1722 and SNP_87 (Dolcetto-specific), the Dolcetto musts/wines represented the target controls and Barbera and Arneis musts/wines represented the non-target controls, and similarly for Arneis-specific SNP_6647 and SNP_16408. For the univocal varietal identification of a must/wine, at least 1 replicate of both cultivar-specific SNPs must be amplified correctly. Using TaqMan® probes, SNP_3356 and SNP_15726 correctly identified Barbera experimental musts/wine at all collection time points, as well as SNP_1722 and SNP_87 in all Dolcetto experimental musts and wines. In some M4 and W technical replicates no amplification was observed (Table 2, Fig. 3). These data confirmed the results obtained in Nebbiolo musts (Boccacci et al., 2020). After malolactic fermentation, TaqMan® genotyping assays showed some amplification problems, probably attributed to the small amount of recovered grapevine DNA. However, with at least two technical replications for sample, it was possible to correctly determine the grapevine genotype from the experimental musts and wine of Dolcetto and Barbera (Table 2). SNP_16408 and SNP_6647 correctly identified experimental Arneis musts at M1 and M3, while no amplification or incorrect allelic calls were observed in wine (Table 2). These amplification problems in Arneis wine are probably linked to the low yield of extracted DNA, as reported for the *VvNCED2* gene. As demonstrated in previous works (Gambino et al., 2022; Song et al., 2024), TaqMan® assays using DNA concentrations lower than 0.5 pg/mL of starting wine are not reliable and may result in a lack of amplification or incorrect allelic identification (Table 1). Overall, considering all target and non-target musts and wine, the SNP_15726, SNP_1722 and SNP_87 assays showed the highest percentages of correct allelic identification (88.4%). Furthermore, the six SNP TaqMan® assays showed a higher percentage of correct allelic calls in the experimental Dolcetto musts and wines (93.3%) than in Barbera musts/wines (86.6%), and significantly higher than in Arneis musts/wines (63.9%) (Table 2). These data demonstrate how the genotype effect can be important in determining the effectiveness of a molecular assay for varietal identification in wines, even in experimental wines vinified in the same winery with the same oenological procedures as in Dolcetto and Barbera. The strong reduction observed in Arneis wines was probably linked to a fermentation without solid parts (skins, seeds and in some case also stalks) and the use of adjuvants, mainly pectolytic enzyme or bentonite, gelatine and silica sol (Gambino et al., 2022).

**Table 2:** Allelic profiles of genotyping assays from Barbera (_B), Dolcetto (_D) and Arneis (_A) musts (M) and wines (W) collected during five and three (for Arneis) experimental winemaking steps. SNPs specific for Barbera (SNP_15726, SNP_3356), Dolcetto (SNP_1722, SNP_87) and Arneis (SNP_16408, SNP_6647) were analysed using high resolution melting (HRM) and TaqMan® probes. Lower-case letters in the allelic profile denote an incorrect call of the genotyping assay; “-” indicates a sample without amplification or incorrect amplification in HRM. For each sample, two independent extractions were analysed (R1, R2).

Must/Wine	Description	TaqMan® probes												High Resolution Melting											
		Barbera				Dolcetto				Arneis				Barbera				Dolcetto				Arneis			
		SNP_15726		SNP_3356		SNP_1722		SNP_87		SNP_6647		SNP_16408		SNP_15726		SNP_3356		SNP_1722		SNP_87		SNP_6647		SNP_16408	
		Alleles		Alleles		Alleles		Alleles		Alleles		Alleles		Alleles		Alleles		Alleles		Alleles		Alleles		Alleles	
		R1	R2	R1	R2	R1	R2	R1	R2	R1	R2	R1	R2	R1	R2	R1	R2	R1	R2	R1	R2	R1	R2	R1	R2
**M1_B**	mashing	**AA**	**AA**	**AA**	**AA**	AA	AA	GG	GG	AG	AG	GT	GT	**AA**	**AA**	**AA**	**AA**	AA	AA	GG	GG	aa	AG	–	–
**M2_B**	end maceration	**AA**	**AA**	**AA**	**AA**	AA	AA	GG	GG	AG	AG	GT	GT	**AA**	**AA**	**AA**	**AA**	AA	AA	GG	GG	AG	AG	–	–
**M3_B**	after AF*	**AA**	**AA**	**AA**	**AA**	AA	AA	GG	GG	AG	AG	GT	GT	**AA**	**AA**	**AA**	**-**	AA	AA	GG	–	aa	aa	–	–
**M4_B**	after MLF**	**AA**	**AA**	**AA**	**-**	AA	gg	GG	–	gg	AG	tt	GT	**AA**	**-**	**-**	**-**	–	–	–	–	–	–	–	–
**W_B**	wine	**AA**	**AA**	**-**	**AA**	AA	AA	GG	GG	AG	gg	GT	tt	**cc**	**cc**	**AA**	**-**	–	–	–	–	–	–	–	–

**M1_D**	mashing	AC	AC	AA	AA	**GG**	**GG**	**AA**	**AA**	GG	GG	GG	GG	aa	–	AA	AA	**GG**	**GG**	**AA**	**AA**	GG	GG	–	–
**M2_D**	end maceration	AC	AC	AA	AA	**GG**	**GG**	**AA**	**AA**	GG	GG	GG	GG	AC	aa	AA	–	**GG**	**GG**	**AA**	**AA**	GG	GG	–	–
**M3_D**	after AF*	AC	AC	AA	AA	**GG**	**GG**	**AA**	**AA**	GG	GG	GG	GG	–	AC	–	AA	**-**	**-**	**AA**	**AA**	aa	aa	–	–
**M4_D**	after MLF**	–	AC	AA	AA	**GG**	**GG**	**AA**	**AA**	GG	GG	GG	GG	cc	–	–	AA	**-**	**-**	**AA**	**AA**	–	–	–	–
**W_D**	wine	AC	cc	AA	ag	**-**	**GG**	**AA**	**AA**	GG	GG	GG	GG	aa	–	–	–	**-**	**-**	**AA**	**AA**	–	GG	–	–

**M1_A**	mashing	CC	CC	AG	AG	AA	AA	GG	GG	**AA**	**AA**	**TT**	**TT**	ac	CC	aa	aa	AA	AA	GG	GG	**AA**	**AA**	**-**	**-**
**M3_A**	after AF*	CC	CC	–	AG	AA	AA	GG	–	**AA**	**-**	**TT**	**TT**	CC	–	–	gg	–	–	GG	–	**-**	**-**	**-**	**-**
**W_A**	wine	CC	–	–	gg	–	–	GG	–	**gg**	**-**	**-**	**gg**	–	–	–	–	–	–	–	–	**-**	**AA**	**-**	**-**

*AF= alcoholic fermentation.

**MLF=malolactic fermentation.

**Fig. 4:**
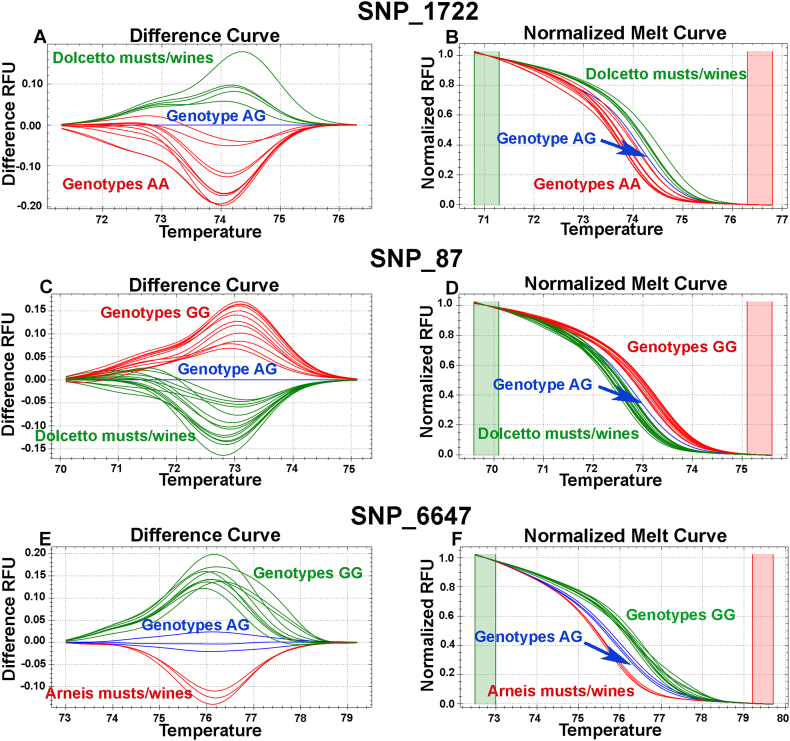
HRM analysis of SNP_1722, SNP_87 and SNP_6647. Normalised melting curves (B, D, F) and difference plots (A, C, E) correspond to two representations of the same data obtained for the three SNPs. Samples from Dolcetto (A–D) and Arneis (E–F) experimental musts and wines were grouped based on the shape of the melting curve into three distinct groups corresponding to the three expected allelic profiles. Genotypes were assigned using a cut-off confidence value of 95%.

The same DNA samples were then analysed with five SNPs using the HRM method; SNP_16408 was excluded due to the problem in the melting curve reported above (Fig. S2). The HRM method did not perform as well as the TaqMan® probes. In musts/wine of Barbera and Dolcetto, the allelic calls were optimal only in M1 and M2. In M3, after alcoholic fermentation, the first amplification problems were observed, and at M4 and W it was not possible to correctly identify the experimental musts/wines. Furthermore, in musts/wine of Arneis, some problems were observed at M1, as well as at M3 and W (Table 2, Fig. 4, Fig. S7). The HRM analyses in the experimental musts and wines highlighted two types of problems, lack of PCR amplification or incorrect amplification, producing non-specific melting curves with melting peaks even 5–6 °C different from those expected for the three possible allelic combinations at each SNP locus (Fig. S8). These differences in melting peaks cannot be attributed to unknown allelic variants, as the nucleotide sequences of the cultivars used in this experiment are well known in the SNP loci considered and no other allelic variants were observed. Samples producing amplicons with non-specific melting peaks were therefore excluded from subsequent analyses so as not to alter the analyses of the melting curves of samples that amplified correctly. As shown with the artificial mix of genomic DNA extracted from the leaf (Fig. 1, Fig. 2), the HRM technique was less performing than the TaqMan® approach. Furthermore, in samples with small quantities of DNA and contaminated by secondary metabolites present in musts and wines, several non-specific amplifications were observed in the HRM which compromised the reliability of the assays. However, HRM has proven effective in detecting grape DNA in the must and wine from Portuguese varieties (Pereira et al., 2017). This could be linked to the analysis of different wines, the use of loci with multiple mutations in the same amplicon and a longer work of fine-tuning the assay. In amplicons with a single SNP mutation, the TaqMan® approach does not require a complex setup and guarantees greater sensitivity and specificity than HRM due to the use of FAM/VIC-labelled probes, which increases the reliability of the technique for SNP marker detection in complex matrices such as musts and wines.

### 3.4 SNP genotyping in commercial wines

SNP genotyping assays were tested in commercial wines of Barbera (1-, 2- and 3-years old), Dolcetto (1- and 2-years old) and Arneis (1-year old). The yield of DNA extracted from all wines was consistent with the previous data reported for the CTAB-based method (Boccacci et al., 2020; Gambino et al., 2022), and the DNA quality determined using the spectrophotometric ratio A_260_/A_280_ was higher than that reported for Nebbiolo wines (Boccacci et al., 2020) (Table 1). Quantification of grapevine DNA with the *VvNCED2* TaqMan® probe confirmed the low levels of DNA in the samples extracted from the wines. For 5 out of 12 commercial wines no amplifications were observed, suggesting that the DNA yield was below the detection limit of the assays. Only from the commercial wine Dolcetto 2022 we obtain a high DNA yield and very high A_260_/A_280_ and A_260_/A_230_ ratios (Table 1). Using TaqMan® probes, SNP_3356 and SNP_15726 (Barbera-specific) correctly identified two Barbera commercial wines from the 2022 vintage, in at least one replicate, while amplification problems were observed in Barbera bottled in 2020 and 2021 (Table 3). The 2021 and 2022 Dolcetto commercial wines were correctly identified by SNP_1722 and SNP_87, but only for Dolcetto2021_b did we not observe any amplification. For Dolcetto2022_a, which showed a very high DNA yield and good quality (Table 1), all six SNPs amplified correctly with unexpected results for a commercial wine (Table 3). The DNA extracted from the commercial wines of Arneis showed amplification problems in Arneis-specific SNP_16408 and SNP_6647; only Arneis 2022_c was correctly identified (Table 3). The SNP genotyping assays based on the TaqMan® probe have shown limitations for the authentication of some commercial wines, as previously reported (Catalano et al., 2016; Boccacci et al., 2020). We determined whether the DNA extracts contained PCR inhibitors by adding an exogenous internal positive control (EIPC). By setting the amplification efficiency to 100% in the controls containing optimal quality DNA extracted from leaves, the amplification efficiency of all commercial wines ranged between 98 and 105%, with no statistical differences from the control, demonstrating that were no PCR inhibitors in the DNA extract. As reported above for experimental musts and wines, a clear difference was observed in the efficiency of the TaqMan® assays based on the type of wine. Four of five commercial Dolcetto wines, two of four commercial Barbera wines and one of three Arneis wines were correctly identified (Table 3). The data from Dolcetto and Barbera wines confirmed that TaqMan® genotyping assays were more effective and sensitive than traditional SSR markers (Baleiras-Couto and Eiras-Dias, 2006; Boccacci et al., 2012; Recupero et al., 2013) for grape identification in commercial wines. DNA extraction from musts and wines produced from Dolcetto grapes appears to be more efficient, guaranteeing greater DNA recovery and better quality which facilitates the genetic traceability of these products. DNA extraction from Arneis musts and wines presents more problems, which could be overcome by developing a more efficient extraction method specific to these wines. The effect of age on the molecular traceability of wine was potentially observed in Barbera; wines bottled in 2022 showed better results than those bottled in 2020 and 2021, although it was not possible to clearly demonstrate this aspect in our experimental plan, in which different wines from different producers were analysed. In fact, wines of the same vintage from different producers showed a great variability of results, as observed for Dolcetto and Arneis (Table 3). As demonstrated for Nebbiolo wines, the effect of the oenological processes used during wine production (processing aids, additives, filtration) has a greater impact than the age of the wine in varietal identification through molecular markers (Gambino et al., 2022; Song et al., 2024).

**Table 3:** Allelic profiles of genotyping assays from Barbera, Dolcetto and Arneis commercial wines. SNPs specific for Barbera (SNP_15726, SNP_3356), Dolcetto (SNP_1722, SNP_87) and Arneis (SNP_16408, SNP_6647) were analysed using high resolution melting (HRM) and TaqMan® probes. Lower-case letters in the allelic profile denote an incorrect call of the genotyping assay; “-” indicates a sample without amplification or incorrect amplification in HRM. For each sample, two independent extractions were analysed (R1, R2).

Wine	TaqMan® probes												High Resolution Melting											
	Barbera				Dolcetto				Arneis				Barbera				Dolcetto				Arneis			
	SNP_15726		SNP_3356		SNP_1722		SNP_87		SNP_6647		SNP_16408		SNP_15726		SNP_3356		SNP_1722		SNP_87		SNP_6647		SNP_16408	
	Alleles		Alleles		Alleles		Alleles		Alleles		Alleles		Alleles		Alleles		Alleles		Alleles		Alleles		Alleles	
	R1	R2	R1	R2	R1	R2	R1	R2	R1	R2	R1	R2	R1	R2	R1	R2	R1	R2	R1	R2	R1	R2	R1	R2
**Barbera 2020**	**-**	**-**	**AA**	**-**	–	AA	–	GG	–	–	–	–	**-**	**-**	**-**	**-**	–	–	–	–	–	–	–	–
**Barbera 2021**	**cc**	**-**	**gg**	**-**	–	AA	–	–	–	–	–	–	**-**	**-**	**-**	**-**	–	–	–	–	–	–	–	–
**Barbera 2022_a**	**-**	**AA**	**-**	**AA**	–	–	–	–	–	gg	–	gg	**-**	**-**	**-**	**-**	–	–	–	–	–	–	–	–
**Barbera 2022_b**	**AA**	**-**	**AA**	**AA**	AA	–	GG	–	AG	–	tt	–	**-**	**-**	**-**	**gg**	–	–	–	–	–	–	–	
**Dolcetto 2021_a**	cc	aa	AA	–	**-**	**GG**	**-**	**AA**	aa	ag	–	–	–	–	–	–	**-**	**-**	**-**	**-**	–	GG	–	–
**Dolcetto 2021_b**	cc	–	–	–	**-**	**-**	**-**	**-**	–	–	GG	GG	–	–	ag	–	**-**	**-**	**AA**	**-**	–	–	–	–
**Dolcetto 2022_a**	AC	AC	AA	AA	**GG**	**GG**	**AA**	**AA**	GG	GG	GG	GG	aa	–	AA	AA	**aa**	**aa**	**AA**	**AA**	GG	GG	–	–
**Dolcetto 2022_b**	–	–	–	–	**-**	**GG**	**AA**	**-**	GG	–	GG	–	–	–	AA	gg	**-**	**-**	**-**	**-**	–	–	–	–
**Dolcetto 2022_c**	cc	–	–	AA	**GG**	**-**	**AA**	**-**	–	–	GG	GG	–	–	–	–	**-**	**aa**	**AA**	**gg**	–	–	–	–
**Arneis 2022_a**	–	CC	gg	–	AA	–	–	–	**AA**	**-**	**gg**	**gg**	–	–	AG	–	–	–	GG	–	**-**	**-**	**-**	**-**
**Arneis 2022_b**	aa	aa	–	aa	AA	–	–	GG	**-**	**gg**	**-**	**gg**	ac	–	–	–	–	–	–	–	**-**	**-**	**-**	**-**
**Arneis 2022_c**	CC	CC	–	AG	AA	AA	GG	–	**AA**	**-**	**-**	**TT**	–	–	aa	–	–	AA	GG	–	**-**	**-**	**-**	**-**

The DNA extracted from the commercial wines was then analysed with the HRM method using five SNPs, as reported above for the experimental musts and wines. The problems highlighted with musts after alcoholic fermentation and in experimental wines also recurred in commercial wines. The HRM technique with these markers did not prove suitable for varietal traceability in wines; in fact, no commercial wines of Barbera, Dolcetto or Arneis were correctly identified (Table 3).

## 4 Conclusion

We identified and validated six new SNP markers specific for three important grapevine genotypes from north-western Italy, Barbera, Dolcetto and Arneis, used in the production of high-quality wines. These SNPs have been successfully used for varietal authentication in musts and wines produced from these genotypes, using TaqMan® assay and HRM analysis for SNP genotyping. The protocol described in this work for the identification of grapes in must and wine can be easily applied to any wine since SNP data are available for many unique genotypes (1493), which include the main wine varieties that can potentially be used for fraudulent mixes. TaqMan® probes represent the most robust method for identifying SNPs and are more efficient than the HRM approach, which has achieved poor results in detecting single SNPs in musts and wines with many non-specific amplifications. The technique probably requires a more laborious set-up than TaqMan® probes and higher quality DNA, but HRM can nevertheless be useful in the presence of more complex mutations, such as INDELS or multiple SNPs in neighbouring sequences, where TaqMan® probes cannot be used (Pereira et al., 2017). The data from the present work showed that grapevine genotypes, the winemaking process and the SNP locus can influence the efficiency of TaqMan® genotyping assays for the correct detection of grapes in wine. However, these variables become decisive for the success of the assay only by analysing low quantities of grape DNA typical of the extracts of some wines; therefore, further efforts are necessary to improve the extraction methods and adapt them to different wines.

## Funding sources

This work was supported by 10.13039/100007571Fondazione Cassa di Risparmio di Torino (10.13039/100011968CRT) project “GrapesInWine: tracciabilità genetica dei vini piemontesi”.

## CRediT authorship contribution statement

**Amedeo Moine:** Investigation, Formal analysis, Data curation, Writing – review & editing. **Paolo Boccacci:** Conceptualization, Supervision, Investigation, Formal analysis, Data curation, Validation, Writing – review & editing. **Camilla De Paolis:** Investigation, Formal analysis, Data curation, Validation. **Luca Rolle:** Conceptualization, Supervision, Writing – review & editing. **Giorgio Gambino:** Conceptualization, Supervision, Funding acquisition, Methodology, Visualization, Writing – original draft.

## Declaration of competing interest

The authors declare that they have no known competing financial interests or personal relationships that could have appeared to influence the work reported in this paper.

## Data Availability

Data will be made available on request.
