# Supplementary material for: TaqMan® and HRM approaches for SNP genotyping in genetic traceability of musts and wines
Source: Curr Res Food Sci. 2024 Feb 22;8:100707. doi: 10.1016/j.crfs.2024.100707 (PMC10912045; doi:10.1016/j.crfs.2024.100707)
Supplement: Multimedia component 2 [file mmc2.pdf]

TaqMan® and HRM approaches for SNP genotyping in genetic traceability of musts and wines

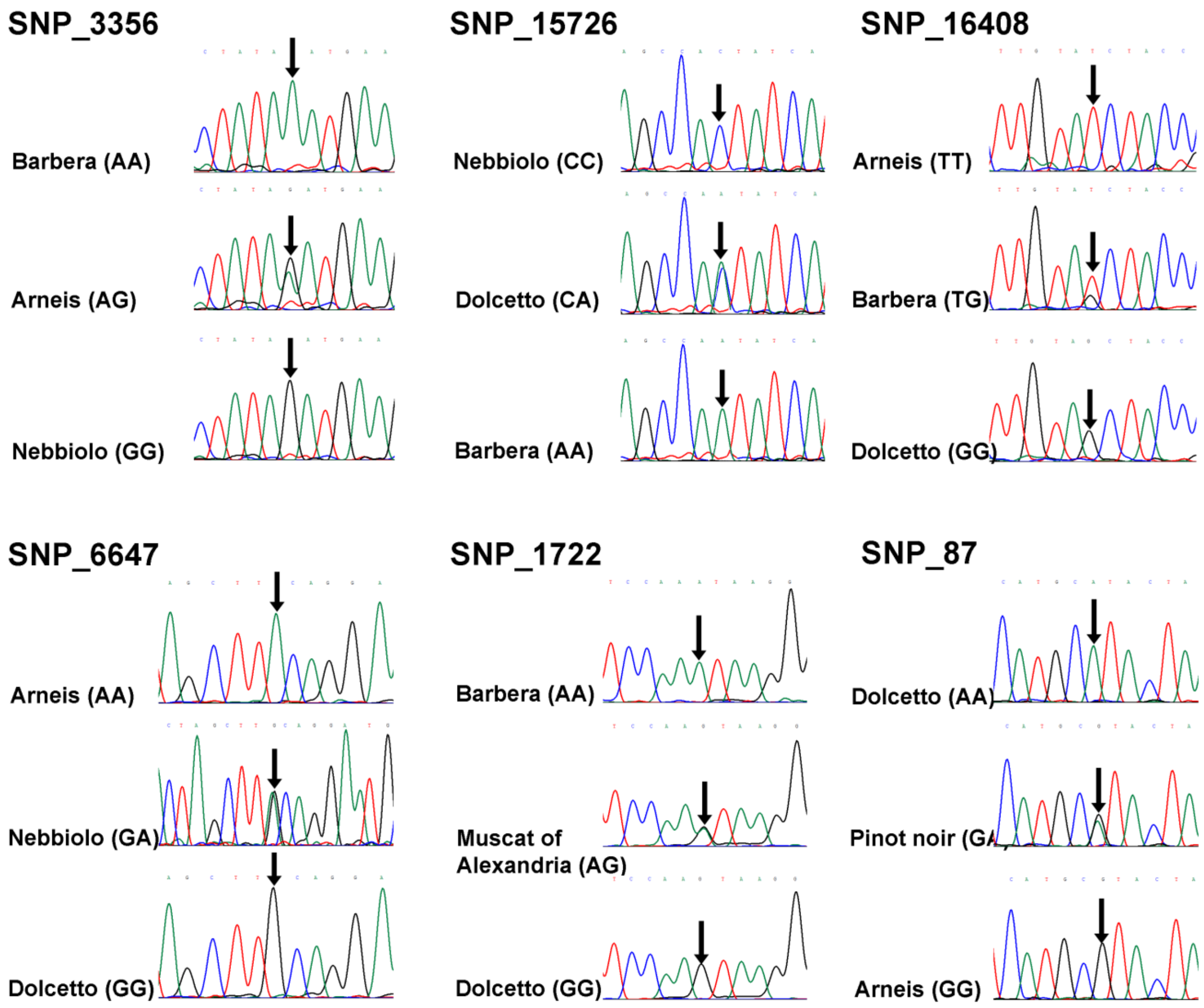

Figure S1. Output of Sanger validation of six SNPs specific for Barbera (SNP_3356, SNP_15726), Arneis (SNP_16408, SNP_6647) and Dolcetto (SNP_1722, SNP_87) selected in this work. The arrows indicate the allelic variants of the SNPs.

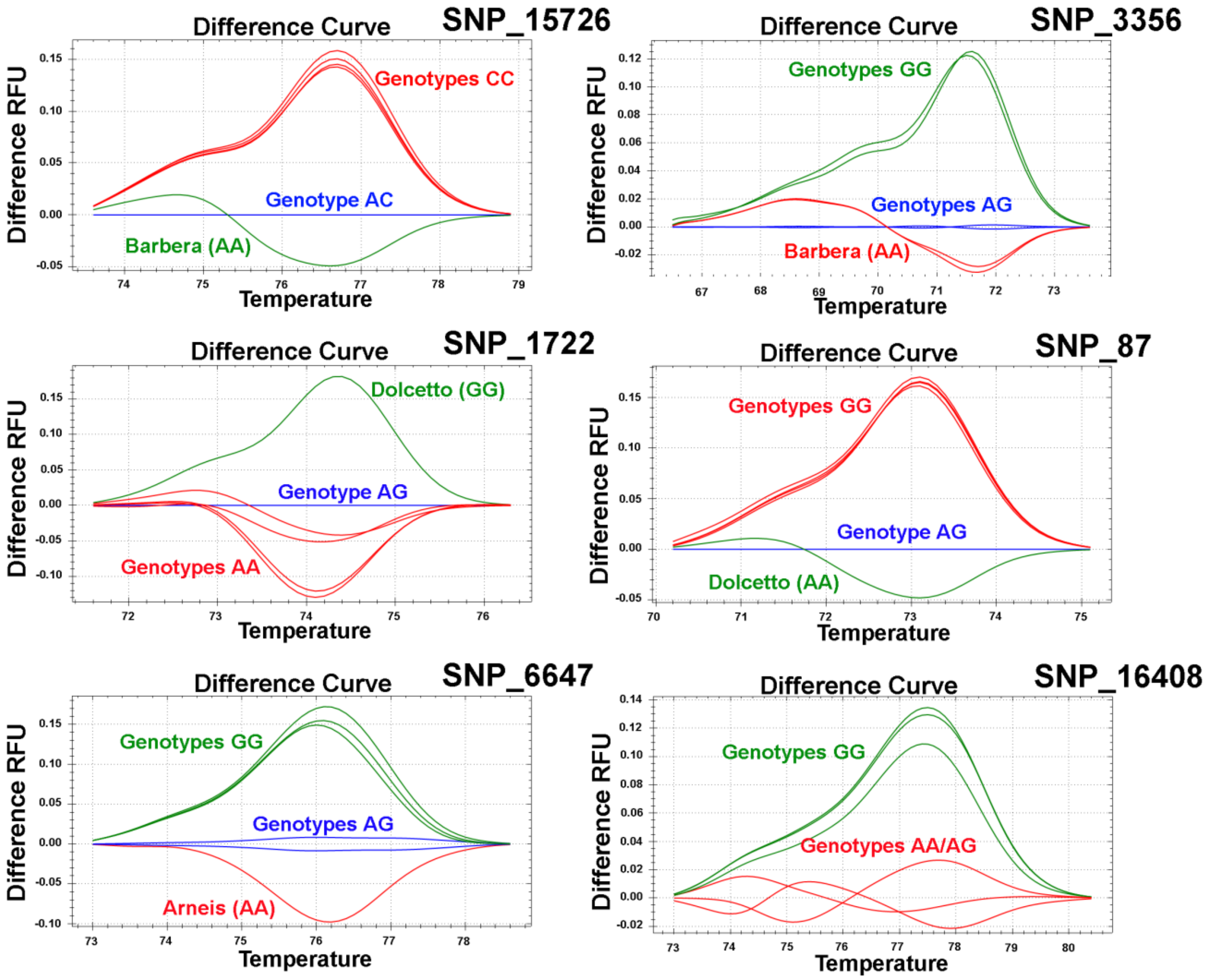

Figure S2. High-resolution DNA melting curve differences for the amplicons produced from SNP_3356, SNP_15726 (Barbera-specific), SNP_16408, SNP_6647 (Arneis-specific), SNP_1722 and SNP_87 (Dolcetto-specific). For SNP_16408, HRM analysis did not distinguish heterozygous (AG) from homozygous (GG) genotypes. All DNA was extracted from leaves. Genotypes were assigned using a cut-off confidence value of 95%.

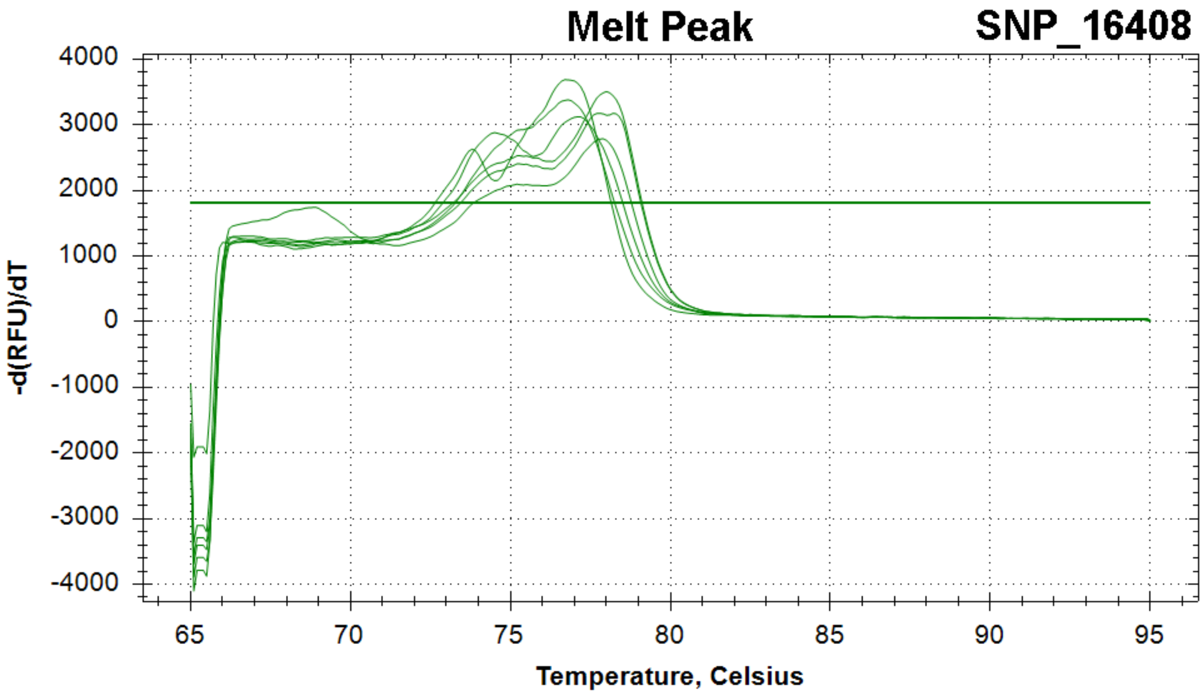

Figure S3. Melting curves of amplicons from locus SNP_16408. The melting curves appear with multiple nonspecific peaks and the locus is not suitable for HRM analysis, as shown in Figure S2.

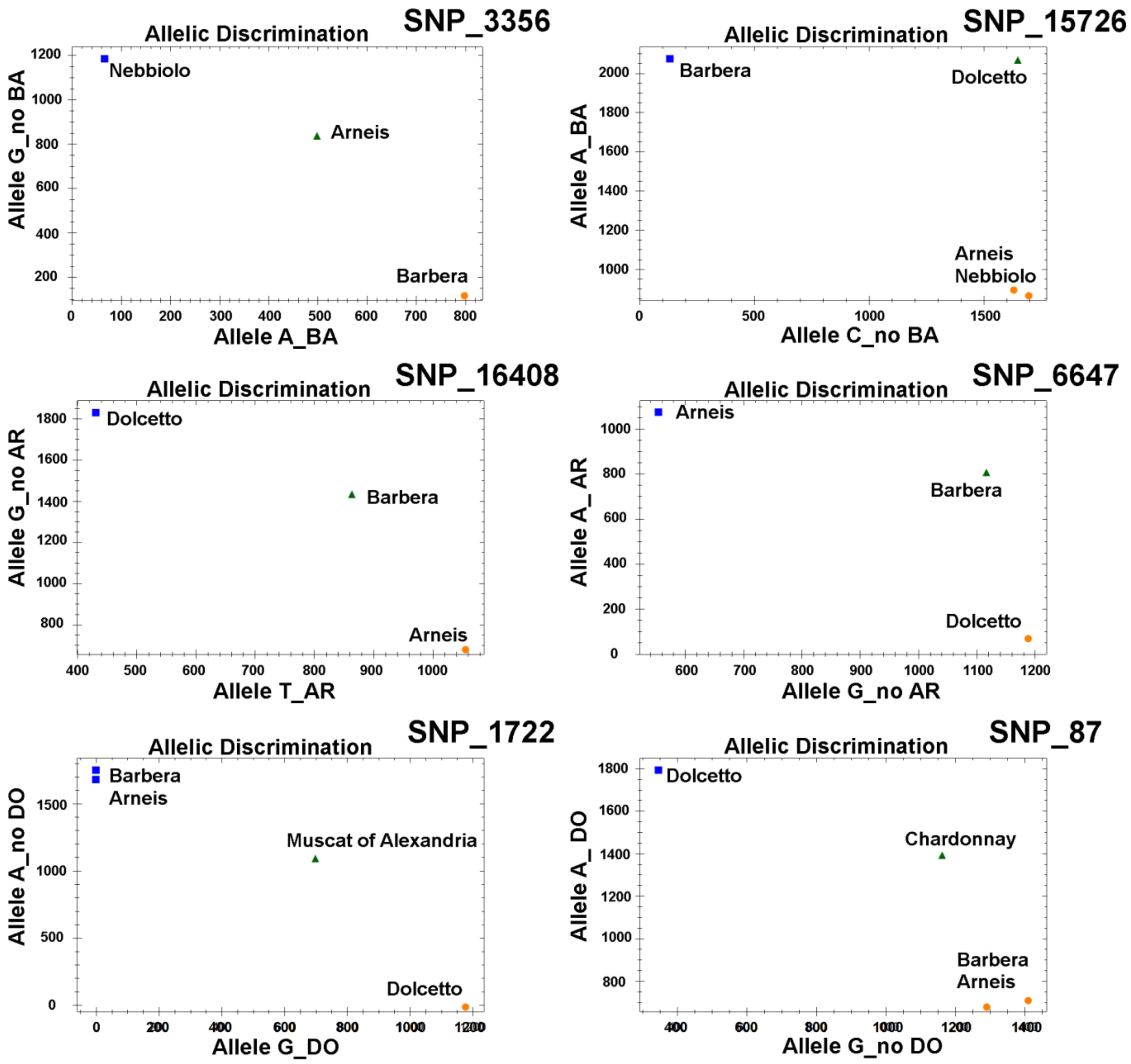

Figure S4. Output of TaqMan® SNP_3356, SNP_15726 (Barbera-specific), SNP_16408, SNP_6647 (Arneis-specific), SNP_1722 and SNP_87 (Dolcetto-specific) genotyping assays. Blue squares and orange points correspond to positive controls for homozygous genotypes; green triangles are positive controls for heterozygous genotypes. DNA was extracted from leaves.

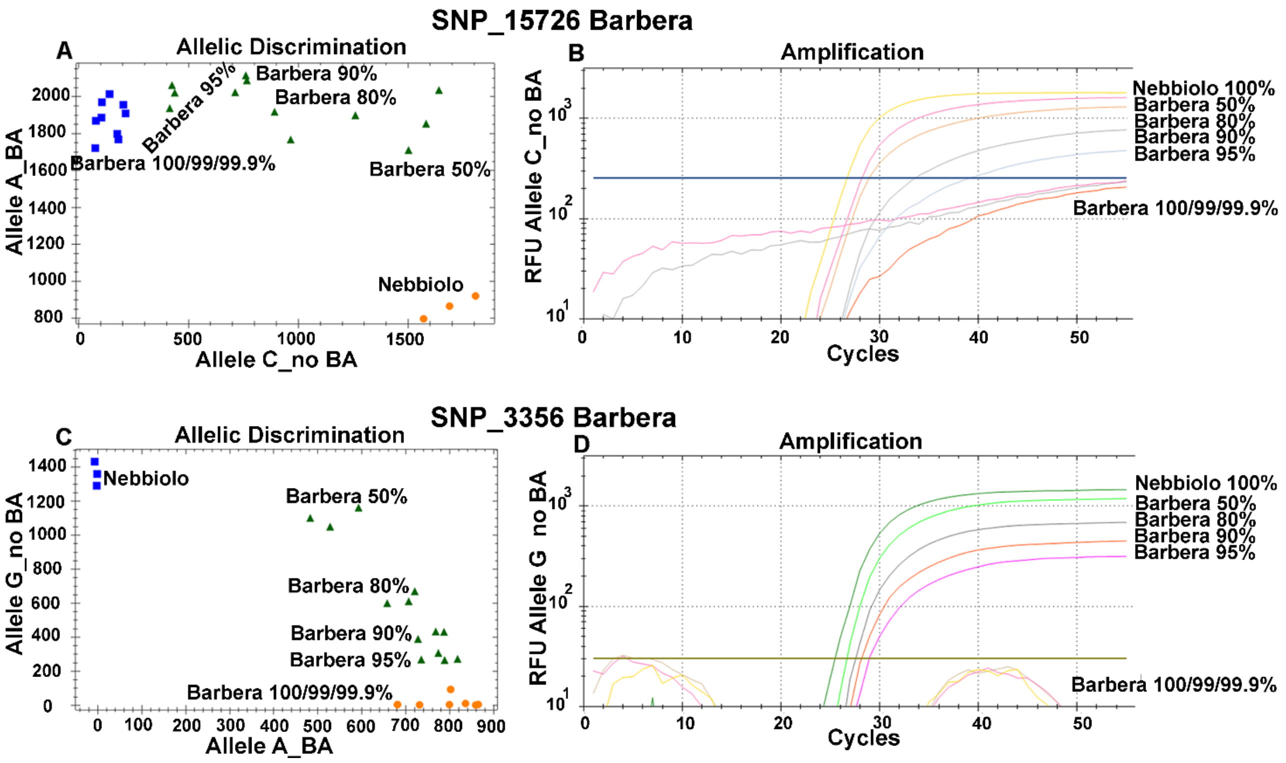

Figure S5. Detection limit of TaqMan® SNP_15726 and SNP_3356 genotyping assays in mixtures of DNA extracted from leaves. Allelic discrimination plots (A, C) and relative fluorescence unit (RFU) of the TaqMan® probes specific for non-Barbera alleles (B, D). Increasing levels of Nebbiolo DNA (from 0.1–50%) were mixed with Barbera DNA. The blue line (B) and yellow line in the amplification plot (D) indicate the RFU level of 100% Barbera, above which it was possible to detect contamination of non-Barbera DNA. For each SNP assay, the detection limit of 5% of non-Barbera DNA mixed in Barbera DNA was determined using three replicates of each sample.

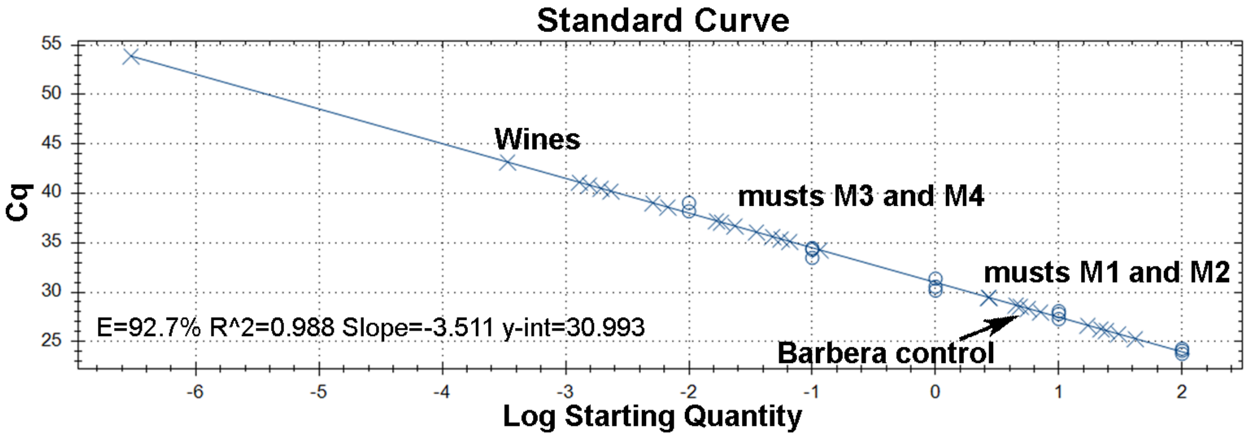

Figure S6 Standard curve of *VvNCED2* TaqMan® probe used to quantify grapevine DNA present in the extracts from experimental musts and wines from Barbera, Dolcetto and Arneis. DNA from Barbera leaves was used as calibrator for the standard curve.

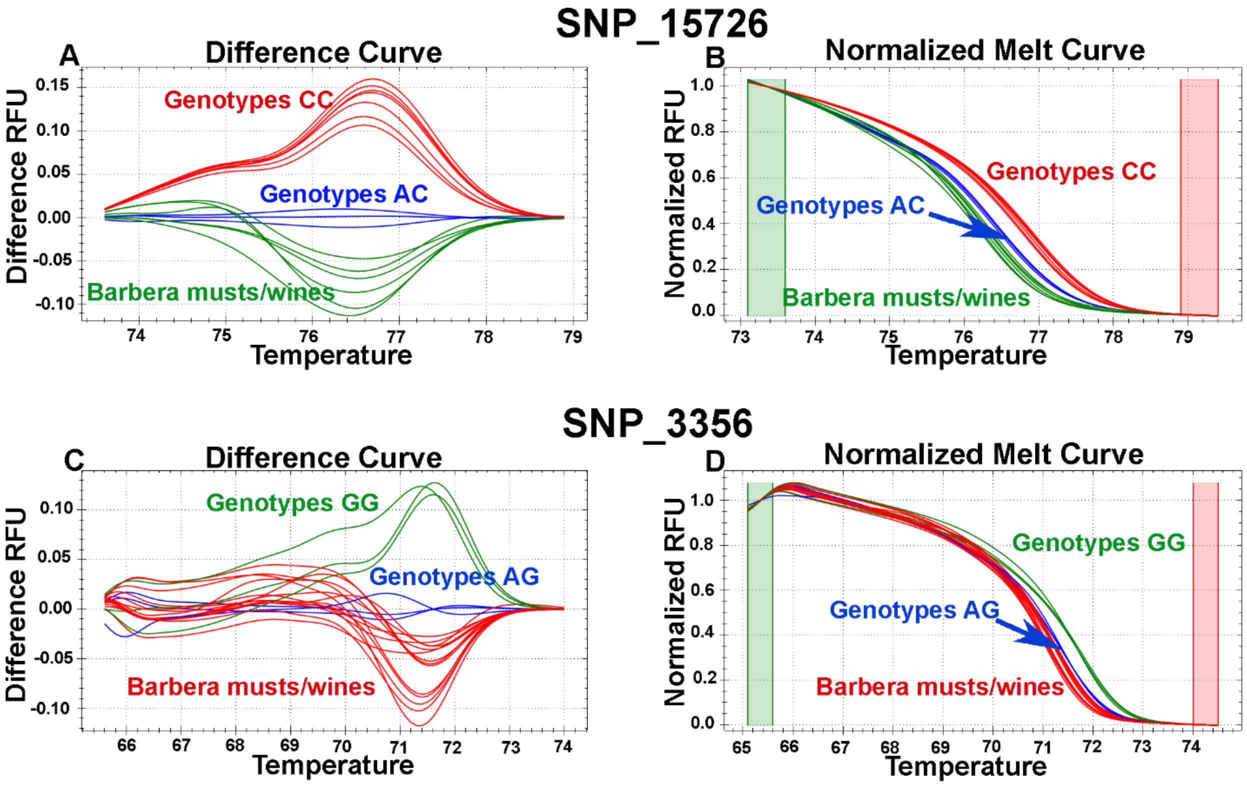

Figure S7. HRM analysis of SNP_15726 and SNP_3356. Normalised melting curves (B, D) and difference plots (A, C) correspond to two representations of the same data obtained for SNP_15726 and SNP_3356. Samples from Barbera musts and wines were grouped based on the shape of the melting curve into three distinct groups corresponding to the three expected allelic profiles. Genotypes were assigned using a cut-off confidence value of 95%.

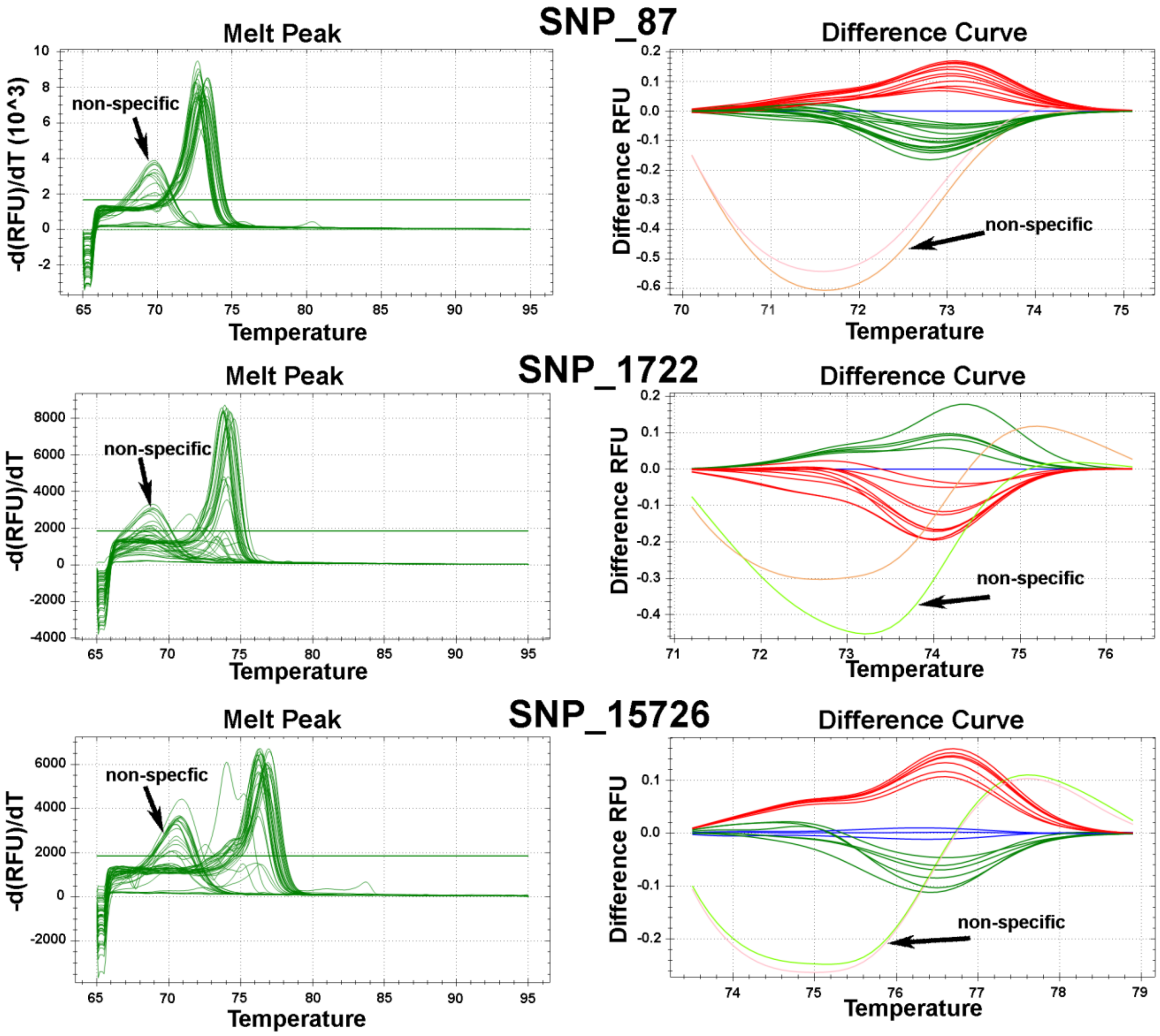

Figure S8. HRM analysis of SNP_87, SNP_1722 and SNP_15726. For each SNP locus, melting curves and difference plots obtained by amplifying the musts and wines of Barbera and Dolcetto were reported. In each analysis, nonspecific amplifications (nonspecific melting peaks) of DNA from musts and wines produced nonspecific melting curves, different from the curves associated with the 3 allelic combinations expected for each locus. Samples with non-specific melt peaks were removed from the analysis. Genotypes were assigned using a cut-off confidence value of 95%.
